# Nanosecond Pulse Electric Field Activated-Platelet Rich Plasma Enhances the Return of Blood Flow to Large and Ischemic Wounds in a Rabbit Model

**DOI:** 10.14814/phy2.12461

**Published:** 2015-07-21

**Authors:** Barbara Hargrave, Francis Li

**Affiliations:** 1 Department of Medical Diagnostics and Translational Science, Old Dominion UniversityNorfolk, Virginia, USA; 2 Frank Reidy Center for Bioelectrics, Old Dominion UniversityNorfolk, Virginia, USA

**Keywords:** Ischemic wound, Nanosecond-pulsed electric field, platelet-rich plasma

## Abstract

Platelet-rich plasma is a therapeutic strategy used for accelerating wound healing of a wide range of tissues through the release of platelet growth factors. Here, we describe a nonchemical, safe method for preparing platelet-rich plasma using nanosecond-pulsed electric fields (nsPEFs) and investigated the effect of this platelet-rich plasma on reperfusion of blood in large skin flap or ischemic hind limb wounds in New Zealand White rabbits. Laser Doppler images of blood flow to the dorsal surface of skin flap wounds or to ischemic hind limb wounds were obtained from wounds treated with 0.9% saline or nanosecond-pulsed electric field prepared platelet-rich plasma (nsPRP). Reperfusion in the skin flap wounds was greater in the nsPRP-treated wounds than in the wounds treated with saline on postoperative days 3 (*P* < 0.001) and 21 (*P* < 0.03). Reperfusion in the ischemic hind-limb treated with nsPRP was greater than in the saline-treated limb on post-operative Day 3 (*P* < 0.001), post-operative week 1 (*P* < 0.025) and post-operative week 4 (*P* < 0.015). In the hind limb ischemic tissue, the number of endothelial cells, collagen, and cells containing vascular endothelial growth factor (VEGF) was greater in the nsPRP-treated tissue. These results demonstrate that nsPRP improves blood flow in large surgical skin wounds and in ischemic wounds.

## Introduction

Platelet-Rich Plasma (PRP) is a platelet-rich concentrate which is activated to release growth factors that strongly influence wound healing. Degranulation of the alpha granules in platelets deposits a concentrated amount of proteins, including growth factors, chemokines, and cytokines, onto the wound that are known to play a role in enhancing healing (Knighton et al. 1986, 1988; Everts et al. 2006). PRP is produced autologously to prevent host versus graft complications. Platelet-rich plasma is emerging as an alternative treatment or as a treatment in combination with other therapeutic methods that are both safe and more cost-effective than many of the presently used wound healing treatments.

The standard clinical platelet activator is bovine thrombin. Though effective, many of the principal actions of thrombin may be harmful to some patients. Thrombin is proapoptotic and proinflammatory, both induced by the generation of reactive oxygen species (ROS) (Lopez et al. 2007; Raivo et al. 2009). There is evidence to show that bovine thrombin interferes with the efficacy of PRP. PRP has the potential to increase the osteo-inductivity of demineralized bone matrix. However, its activation with bovine thrombin immediately prior to implantation significantly inhibits this activity (Margolis et al. 2001). To overcome the problems associated with exposing the patient to thrombin-activated PRP, we alternatively activated platelets by exposing them to nanosecond-pulsed electric fields (nsPEF) (Schoenbach et al. 2007; Zhang et al. 2008). These ultrashort, intense electrical pulses activate the platelets and create microscopic pores in the platelet membranes through which growth factors, cytokines, chemokines, and other proteins may permeate. In this way, we obtain a nonchemically activated PRP, nsPEF-activated PRP (nsPRP), which does not pose the systemic risk of an allergic reaction and other untoward effects associated with chemical activators, such as thrombin. We have reported that nsPRP reduces ROS production in vitro and improves left ventricular heart function after myocardial infarction (Hargrave and Li 2012)^.^In addition, we have reported that in the Langendorff rabbit heart model left ventricular function is improved in globally ischemic/reperfused hearts treated with nsPRP (Hargrave 2014).

Recombinant human platelet-derived growth factor-BB isomer (rh-PDGF-BB) has also been used to enhance wound healing (Marx 2001). While growth factors such as rh-PDGF-BB is of human origin, it is foreign to the patient being treated and may be antigenic. The autologous nature of PRP makes it more akin to the natural healing process, and exposes the wound to multiple growth factors all of which may play a role either directly or indirectly in enhancing wound healing.

One hallmark of successfully healing a wound is a significant return of blood flow to the injured site (Zografos et al. 1992). In this study, we investigated the effect of nsPRP on the return of blood flow to the wounded site in two different wound models. One wound was a large surgical wound created on the dorsal surface of the New Zealand White (NZW) Rabbit. The second wound was an ischemic wound created in the right hind limb of the NZW rabbit. The focus of this study is to show that nsPRP is effective in enhancing the return of blood flow to the wound.

## Materials and Methods

We used a total of 38 animals in this study. The use of animals in this study was conducted in an AAALAC accredited facility and in compliance with the animal welfare act and other federal statutes and regulations pertaining to animals. All procedures outlined in this study were approved by the Old Dominion University’s animal care and use committee.

### Nanosecond pulse electric field (nsPEF) and the pulse generator

Nanosecond pulse electric fields are ultrashort, high-voltage-pulsed electric fields that effect intracellular as well as extracellular membranous structures and functions (Schoenbach et al. 2007). Nanosecond pulses convey intense, high power, but low-energy electric fields. During platelet activation, nsPEFs charge the platelet membrane and create pores without inducing permanent damage (Schoenbach et al. 2007; Zhang et al. 2008). The 300 ns pulse generator used for these studies has been previously described (Schoenbach et al. 2007; Zhang et al. 2008). The electrical pulses were applied to PRP in sterile aluminum electroporation cuvettes having an electrode gap of 0.4 cm and an area of 1 cm^2^. The shape and amplitude of the pulse voltage was monitored using a 500 MHz oscilloscope. For the generation of PRP, one milliliter of PRP in the presence of 10 mM CaCl_2_ was exposed to five pulses at an electric field of 30 kV/cm.

### Preparation of platelet rich plasma (PRP)

Hematological analysis of rabbit blood used to prepare nsPRP was performed using a HESKA Diagnostic System, Switzerland. The PRP had an average platelet count of 295 × 10^3^/μL. The Harvest Technology System concentrator (Harvest Technologies, Plymouth, MA) concentrated the platelets in the whole blood 4–7 times providing a platelet concentrate between 1180 × 10^3^/μL and 2065 × 10^3^/μL of platelets.

PRP was prepared per the Harvest Technologies protocol. Briefly, 60 mL of blood was withdrawn from a donor rabbit using a sterile syringe containing 6 mL of ACD-A anticoagulant. A SmartPRep®-2 Platelet Concentrate System and a sterile processing disposable pack (Terumo Cardiovascular Systems, Ann Arbor, MI) was used to prepare PRP. The processing disposable was centrifuged for 14 min to separate the blood components from the plasma. PRP was prepared by resuspending the platelet concentrate with platelet poor plasma to a final volume of 7 mL.

### Preparation of nsPRP Supernatant

Platelets were concentrated as described above. For the generation of PRP, one milliliter of PRP in the presence of 10 mM CaCl_2_ was exposed to five pulses at an electric field of 30 kV/cm. To prepare a PRP supernatant the nsPRP was removed from the cuvette and placed into a sterile 1 mL Eppendorff tube for 10 min at room temperature. The Eppendorff tube was then centrifuged at 14,000 rpms for 10 min. The supernatant was removed and placed into an Alzet Osmotic Pump (Durect Corporation, Cupertino, CA) prior to insertion of the pump into the wound. The remaining pellet was discarded.

### Measurement of blood flow to the wound using a laser Doppler imager

A Moor Laser Doppler Imager (LDI) (Moor Instruments, Wilmington, DE) was used to measure the total local microcirculatory blood perfusion on the dorsal surface of the skin flap wound and in the hind limb ischemic wounds. The technique is based on the emission of a beam of laser light carried by a fiber-optic probe. The light is then scattered and partly absorbed by the tissue being studied. Light hitting moving blood cells undergoes a change in wavelength (Doppler shift) while light hitting static objects is unchanged. The skin flap wound area of the rabbit was imaged using the LDI prior to surgery at Day 0, immediately after surgery, and on postoperative days 2, 7, and 14. For quantitative analysis, the surgical area was integrated to create a region of interest (ROI). The 3 × 8 cm surgically created skin flap wound was arbitrarily divided into a distal, medial and proximal segments for analysis. We report data from all three segments of the wound. A statistical table associated with the ROI was generated by the imaging software (Moor Instruments, Wilmington, DE). The unit of measurement is an arbitrary perfusion unit. Day 0 prior to the surgery was used as a baseline for each animal. In addition, Day 0 after the surgery was used as postoperative baseline for each animal. The percentage of pre-surgery baseline and percentage of postoperative baseline were used for evaluation.

### Surgical wound created on the dorsal surface of the New Zealand white rabbit

New Zealand White Rabbits (Harlan, Inc, Frederick, Maryland) were sedated with a combination of xylaine (5 mg/kg) and ketamine (25 mg/kg) delivered intramuscularly. General anesthesia was induced using isoflurane in oxygen (5 L/min for 1 min then 1.5 L/min for the duration of the procedure). The dorsal surface was shaved and cleansed with 70% ethanol and chlorhex-Q scrub. Once a surgical plane of anesthesia was established, a 3 × 8 cm skin flap wound was created on the dorsal surface of the animal. NsPRP or 0.9% saline in a volume of 1 mL was placed on the wound and the flap sutured to its original position, using 4–0 silk interrupted stitches. We focused our analysis on the distal segment of the wound since this segment had the greatest degree of blood flow disruption.

### Creation of the ischemic hind-limb wound

New Zealand White Rabbits (Harlan, Inc, Frederick, Maryland) were sedated with a combination of xylazine (2 mg/kg) and ketamine (25 mg/kg) delivered intramuscularly. General anesthesia was induced using isoflurane in oxygen (5 L/min for 1 min then 1.5 L/min for the duration of the procedure). To investigate the effect of nsPEF-activated PRP on wound healing in the presence of ischemia, an incision was made on the medial thigh of the right hind limb. Ischemia was created by the removal of a 2 cm segment of the femoral artery. This was viewed as the “wound” to that limb. In this study, we delivered nsPRP supernatant continuously to the wound. An Alzet Osmotic pump (Durect Corporation, Cupertino, CA) measuring 3 × 0.7 cm, was placed subcutaneously in the right thigh of the hind limb parallel to the ischemic region of the wound. The flow rate of the pump was 0.25 μL/h. The pump was filled with 250 μL of either PRP supernatant or saline with a duration of release of 28 days. In this study, we measured blood flow in the wound area on day 0 before and after surgery, followed by postoperative days 3, 7, 14, 21, and 28 using Laser Doppler Imaging.

### Immunohistochemistry

Wound tissue was placed in 10% formalin and sent to an outside vendor for paraffin embedding and processing. Slides containing tissue from the area of interest of the hind-limb ischemic wound were processed as follows: washed in xylene twice, once in 100%, 95%, 0%, and 50% ethanol for 1 min each, then washed in deionized water for 5 min. Three percent hydrogen peroxide was added to each slide, and incubated at room temperature for 10 min then washed again. For antigen retrieval, 150 mL of a citrate buffer was prepared and placed into a container containing the slides in a tissue rack. The container was placed heated in the microwave for 3 min 4 times. The primary antibodies: anti-collagen, VEGF, and CD-31 were used at a dilution of 1:100 (Abcam, Cambridge, MA). The secondary antibody- TRITC red rhodamine (ThermoFisher Scientific, Grand Island, NY) was used at a dilution of 1:125. The SlowFade Gold AntiFade reagent with DAPI (Molecular Probes, Grand Island, NY) was used as a nuclear stain.

### Statistical analysis

The Laser Doppler Imager data were analyzed using an analysis of variance corrected for repeated measures and stated as the mean ± standard deviation. The Tukey and Student-Newman–Keuls post hoc tests were used to determine which groups were statistically different from each other. Where applicable, data are stated as the mean ± SD.

## Results

### 3 × 8 cm skin flap wound

**Figure 1:**
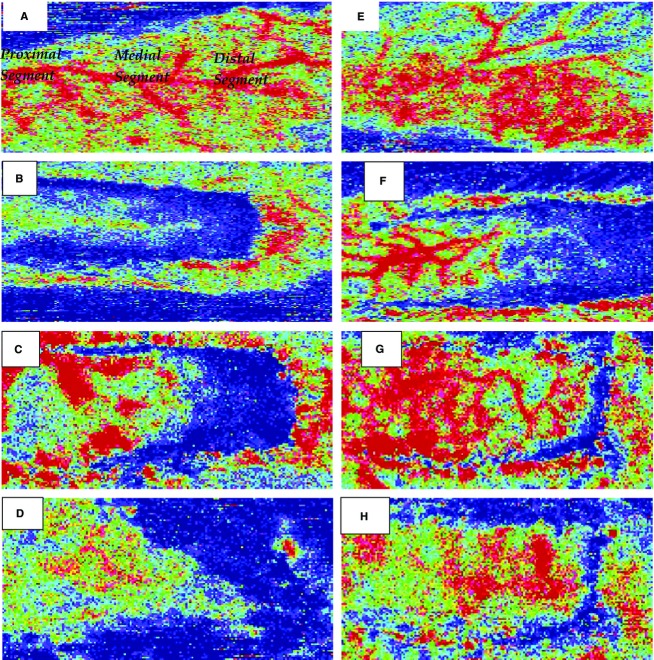
Representative Laser Doppler image of blood flow and its return to the dorsal surface of skin flap wounds treated with 0.9% saline (A through D) or nsPRP (E through H). The blue color within the 3 X 8 wound area is indicative of a lack of blood flow. The red color indicates the presence of blood flow.

Seventeen animals were studied. Figure1, panels A–H show a representative Laser Doppler Image of blood flow and its return in the skin flap wounds treated with 0.9% saline or nsPRP.

**Figure 2:**
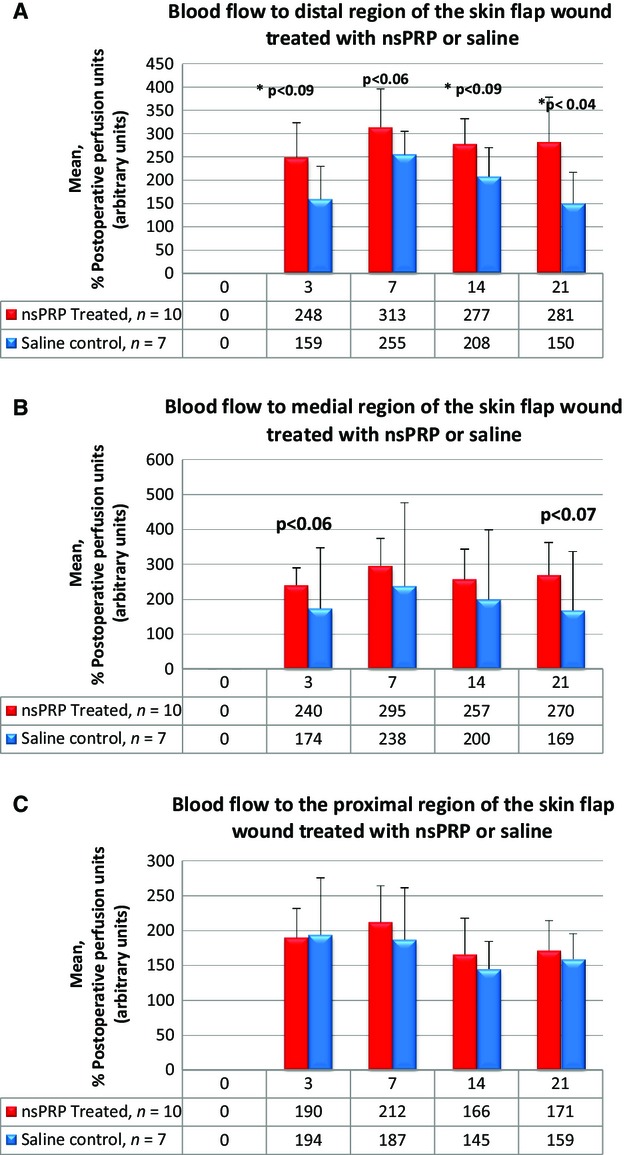
Quantitative analysis of blood flow to the distal (A), medial (B) and proximal (C) segments of the wound. The data were normalized to 100% of postoperative perfusion. Data are stated as mean ± SD. nsPRP = nanosecond pulsed electric field activated PRP; saline control = 0.9% saline; 0 = day of surgery; 3 = days postoperative; 7 = days postoperative; 14 = days postoperative; 21 = days postoperative.

Figures2A–C show the quantitative analysis of blood flow in the distal, medial, and proximal segments of the 3 × 8 skin flap wound. Reperfusion was significantly greater in the nsPRP-treated wounds than in the wounds treated with a saline control on postoperative days 3 (*P* < 0.001) and 21 (*P* < 0.04). The data were normalized to 100% of the postoperative perfusion. PRP prepared, using nsPEFs significantly enhanced the return of blood flow to the distal segment of the skin flap wound in the NZW rabbit. The results were the same when we normalized the data to 100% of the preoperative perfusion (data not shown).

These data suggest that the PRP prepared using nsPEFs enhances the return of blood flow to the distal segment of the skin flap wound in the NZW rabbit. It is the distal segment of the wound that experiences the greatest degree of blood disruption.

### Hind limb ischemic wound

**Figure 3:**
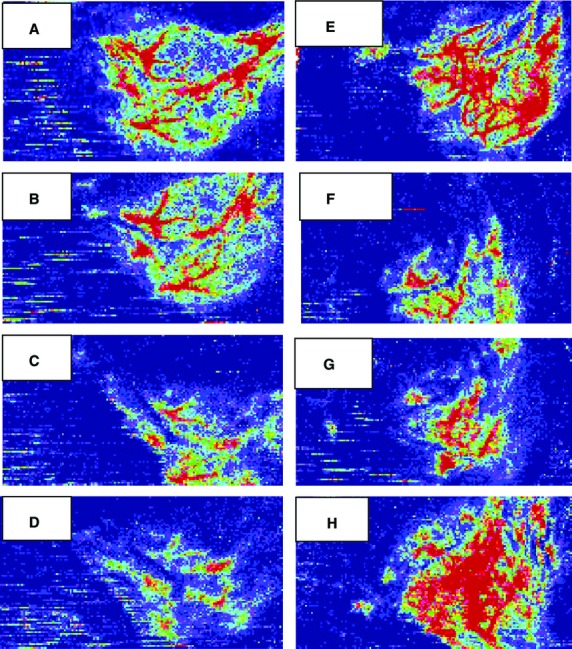
Representative Laser Doppler images of blood flow and its return to the ischemic hind limb wounds treated with 0.9% saline (A through D) or nsPRP (E through H). The blue color within the wound area is indicative of a lack of blood flow. The red color indicates the presence of blood flow.

**Figure 4:**
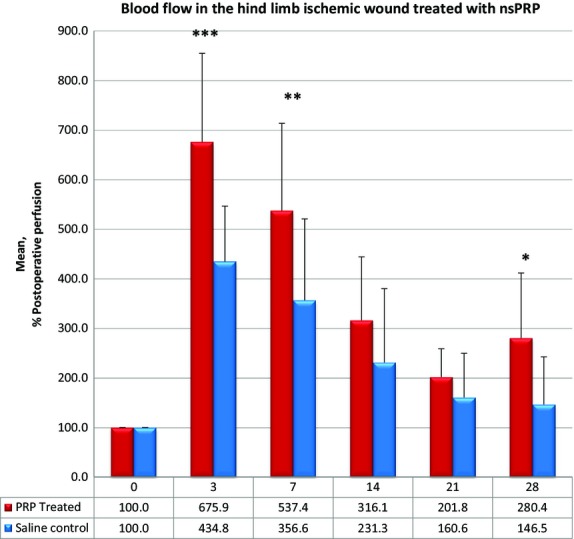
Quantitative analysis of blood flow to the ischemic hind limb wound. The data were normalized to 100% of postoperative perfusion. Data are stated as mean ± SD. 0 = day of surgery; 3, 7, 14, 21 and 28 = days postoperative.

We studied 21 animals in this experiment. Figure3, panels A–H show a representative Laser Doppler Image of the return of blood flow in ischemic hind limb wounds treated with 0.9% saline or nsPRP supernatant. Figure4 shows the quantitative analysis of blood flow to the ischemic hind limb in wounds treated with nsPRP supernatant (n = 10), or 0.9% saline (*n* = 11). The nsPRP supernatant was prepared and the osmotic pump filled as previously described. Reperfusion in the hind-limb treated with nsPRP supernatant was significantly greater than in the saline-treated limb on postoperative Day 3 (*P* < 0.001), post-operative week 1 (*P* < 0.025), and postoperative week 4 (*P* < 0.015). The data were normalized to 100% of the postoperative perfusion. The results were the same when we normalized the data to 100% of the preoperative perfusion (data not shown).

### Immunohistochemistry of Hind-limb Wound

We analyzed the ischemic hind-limb wounds for the presence of selected vascular markers. Table1 summarizes the number of cells per unit area staining positive for the various vascular markers in tissues treated with nsPRP or saline.

**Table 1:** Summary of the number of cells per unit area staining positive for vascular markers or collagen

	Cells/unit area staining positive	
	nsPEF PRP (treated)	Saline (control)
CD-31	17	2
VEGF	30	0
Collagen	27	0

**Figure 5:**
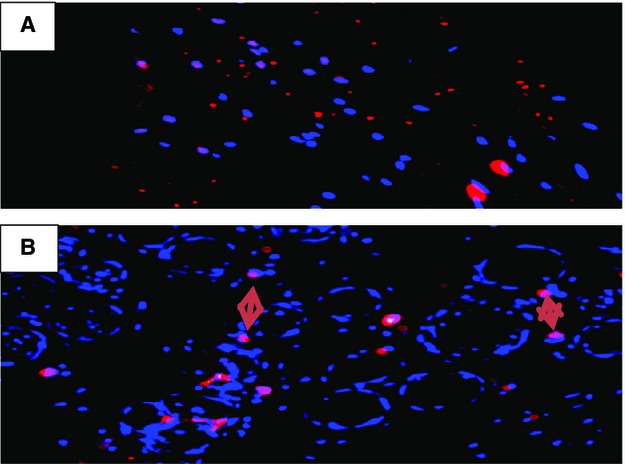
Immunohistochemical analysis of rabbit hind limb ischemic tissue treated with 0.9% saline (A) or nsPRP (B). The arrows indicate the presence of endothelial cells in the nsPRP treated tissue.

To ascertain whether angiogenesis was associated with the increase in blood flow in the nsPRP-treated wounds, we used the vascular marker CD-31. This marker was used to demonstrate the presence of endothelial cells in tissue sections. Figure5 shows that endothelial cells were more prevalent in the nsPRP-treated wounds than in the wounds treated with saline.

**Figure 6:**
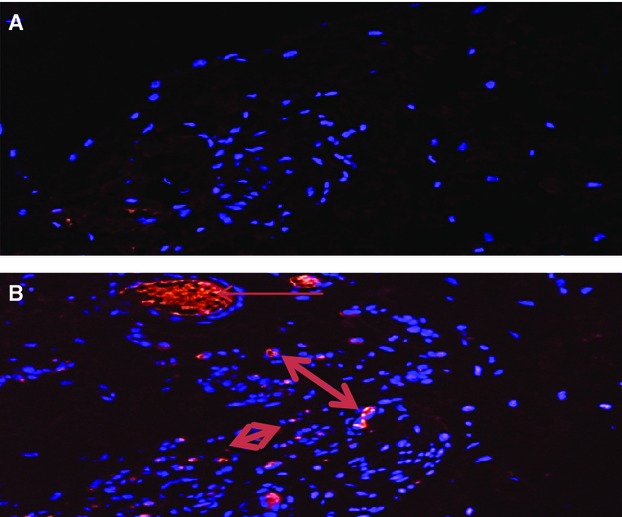
Immunohistochemical analysis of rabbit hind limb ischemic tissue treated with 0.9% saline (A) or nsPRP (B) and stained for the presence of collagen. The arrows indicate cells in the nsPRP treated tissue staining positive for collagen.

**Figure 7:**
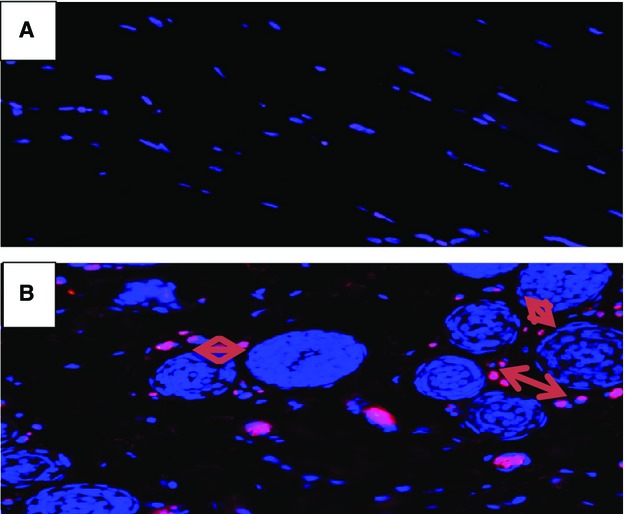
Immunohistochemical analysis of rabbit hind limb ischemic tissue treated with 0.9% saline (A) or nsPRP (B) and stained for the presence of vascular endothelial growth factor (VEGF). The arrows indicate cells in the nsPRP treated tissue staining positive for VEGF.

We also analyzed the tissue for changes in collagen using a collagen marker (Fig.6) and changes in VEGF (Fig.7). There was a greater degree of collagen and VEGF in the ischemic limbs treated with nsPRP than in the hind-limbs treated with saline.

Wounds treated with nsPRP stained positive for markers indicative of angiogenesis and collagen synthesis to a greater extent than the wounds treated with saline. It should be noted, however, that the background appeared greater in the saline micrograph, but not in the PRP-treated tissue and that there was greater nonspecific staining. The rationale for this difference is unclear since all slides were treated using the same protocol and exposed to the same number of washes.

## Discussion

The purpose of this study was to show nsPRP as an effective nonchemical agent in enhancing blood flow to wounds. NsPRP is a safe and cost-efficient method to treat wounds. There are several studies supporting the use of PRP to enhance healing of wounds in general and difficult wounds in diabetic patients (Krupski et al. 1991; Margolis et al. 2001; Cullinane et al. 2002; McAleer et al. 2006; Pietramaggiori et al. 2006; Salemi et al. 2008; Gomez-Caro et al. 2011; Jl et al. 2012; Sakata et al. 2012 Apr; Sönmez et al. 2013 Apr; Kontopodis et al. 2015). In one study, PRP was reported to successfully heal a chronic lower extremity wound after failed attempts at a living skin graft application (Zografos et al. 1992). The combination of adipose tissue and PRP was found by some researchers to enhance graft adherence (Cullinane et al. 2002). In a respective cohort study estimating the effectiveness of platelet foot ulcers, the researchers found that the relative risk for a wound to heal after treatment was 95% when compared to the standard of care (Margolis et al. 2001). Another retrospective longitudinal study by Sakata et al. reported good wound healing and a low amputation rate in patients with diabetes mellitus and ischemia treated with supportive care and PRP (Sakata et al. 2012 Apr). On the other hand, there are also studies that do not support the use of growth factor therapy. Krupski et al. found that wounds treated with a wound healing formula – a mixture of growth factors including PDGF, PF4, TGF-*β*, PDEGF, and PDAF healed no better than traditional therapy (Krupski et al. 1991). Lacci et al. suggest that the presence of confounding variables in the various patients may account for the discrepancy. These variables include differences in diabetic patient characteristics, initial wound area, depth and duration (Lacci and Dardik 2010). It is also possible that the method used for platelet-rich plasma preparation and the platelet count may vary, and therefore yield different results.

We have shown in this study that when the platelet count is relatively constant, and the platelets are concentrated to approximately the same degree (4–7 times baseline platelet count in whole blood) that (1) reperfusion in nsPRP-treated wounds was significantly greater than in the saline-treated wounds; and (2) nsPRP-treated wound have markers present indicating angiogenesis. While the ischemic hind-limb wound was continually exposed to nsPRP for 28 days via the Alzet Osmotic Pump is unclear whether an infusion of nsPRP is required for it to be effectiveness versus a single intra-wound injection. In this study, we elected to continually expose the ischemic hind-limb wound to nsPRP because of the severity of the wound. However, the skin flap wounds received a onetime treatment and blood flow was restored to these wounds to a degree that was significantly greater than what was observed in the control animals. The immunohistochemical data presented in this study was obtained from the more severe ischemic hind limb wounds and showed that wounds treated with nsPRP had a greater presence of endothelial cells, collagen and VEGF compared to tissue treated with saline. These data suggest that there was greater angiogenesis in the nsPRP-treated tissue and are supported by the work of Sonmez et al. who concluded from their studies that the angiogenic effects of PRP may be potentiated by ischemia (Sönmez et al. 2013 Apr).

We did not use bovine thrombin-activated platelets as a control in this study since it was not our intention to compare or study the effects of bovine thrombin. However, in subsequent experiments treating the infarcted tissue in the rabbit heart with PRP prepared using nsPEFs or bovine thrombin the nsPRP was associated with greater left ventricular function than hearts treated with bovine thrombin PRP. This was not observed in the hearts treated with vehicle (bovine serum albumin) (Hargrave 2014). Also, we previously compared a platelet-rich plasma supernatant prepared using bovine thrombin to a platelet-rich plasma supernatant using nsPEF and found that there was little or no endogenous antioxidants (i.e., catalase and superoxide dismutase) in the thrombin-activated platelets compared to the nsPEF activated platelet-rich plasma (Hargrave 2014). This is consistent with the report by Han et al. showing that PRP prepared using bovine thrombin inhibited induction of osteo-inductivity of demineralized bone matrix (Han et al. 2009).

NsPEF activation of platelets for the preparation of platelet-rich plasma is both safe and effective. It is nonchemical, so there are no adverse systemic effects that tend to be associated with most chemical activators. Using immunohistochemistry, we have shown that nsPRP increased vascular and collagen markers that are designed to produce vessel formation and collagen synthesis, respectively, thereby providing a mechanism for the enhanced blood flow observed in the PRP-treated wounds. In summary, we investigated the effect of nsPRP on large skin flap wound and on ischemic wounds, and found nsPRP to be effective in enhancing blood flow to the injured tissue.
